# Nutritional content of vitamin and mineral supplements aimed at children in the peruvian market: analysis of compliance with recommendations

**DOI:** 10.17843/rpmesp.2025.421.14256

**Published:** 2025-03-17

**Authors:** Lorena Saavedra-Garcia, Anna Magdalena Gawlas, Antonella Quiroz-Macukachi, Hans Genmayel Donayre-Huamán, Raquel Idelsa Basurco-Olazabal, Jamee Guerra Valencia

**Affiliations:** 1 Functional Nutrition Research Group, Nutrition and Dietetics Degree Program, Faculty of Health Sciences, San Ignacio de Loyola University, Lima, Peru. San Ignacio de Loyola University Functional Nutrition Research Group, Nutrition and Dietetics Degree Program Faculty of Health Sciences San Ignacio de Loyola University Lima Peru; 2 Medicine Degree, Faculty of Health Sciences, San Ignacio de Loyola University. Lima, Peru. San Ignacio de Loyola University Medicine Degree Faculty of Health Sciences San Ignacio de Loyola University Lima Peru; 3 Faculty of Health Sciences, Universidad Privada del Norte, Lima, Peru. Universidad Privada del Norte Faculty of Health Sciences Universidad Privada del Norte Lima Peru

**Keywords:** Dietary Supplements, Child, Vitamins, Minerals, Recommended Dietary Allowances, Latin America

## Abstract

We described the nutritional content of vitamin and mineral dietary supplements for children on the Peruvian market and their compliance to the recommended dietary intake. A cross-sectional study was carried out with the information declared on the packaging of 34 products sold in pharmacies during 2022. The daily doses of each micronutrient were calculated according to the manufacturer’s instructions; we also estimated the compliance with the recommended dietary allowance (RDA) and the tolerable upper intake level (UL) by age group. The most common micronutrients were vitamins C (n=23), D (n=22), A (n=16), zinc (n=15) and B6 (n=14). In all groups, vitamin D, thiamine, riboflavin, folate, vitamin B12 and vitamin C exceeded the RDA. Zinc and copper also exceeded the recommendations in some groups. Vitamin A and folate most frequently exceeded the UL. Our findings highlight the need for greater health surveillance.

## INTRODUCTION

Globally, the dietary supplement industry reached USD 454.55 billion in 2021, with growth in several regions ^1^. In Latin America, the supplement market grew from 3% in 1999 to 7% in 2017, with vitamins and minerals being the most consumed in Chile, Brazil, and Peru ^2^. This growth is also seen in the pediatric population, where the global market is estimated to grow from USD 2.5 billion to USD 4 billion by 2034 ^3^.

Attractive packaging and marketing aimed at children ^4^, together with their availability without prescription, can create a perception of safety and encourage their use without supervision. Furthermore, the lack of specific regulations on their content allows manufacturers to freely use ingredients and recommended amounts, leading to variability in composition ^5^^,^^6^, which, in turn, can lead to doses exceeding the tolerable upper intake level (UL) ^6^^,^^7^.

Previous studies have found that the micronutrient contributions indicated on dietary supplement labels often exceed the recommended daily allowances (RDAs) ^5^ and even the ULs ^6^^,^^7^. Furthermore, when dietary intake and supplements are combined, excess intake is more common ^8^, suggesting a risk of overexposure.

In the United States, up to 31.8% of hospitalizations associated with dietary supplements are attributed to adverse effects and 10.2% to excessive doses ^9^, which can cause multiple adverse events such as nausea, anorexia, and hepatotoxicity, depending on the nutrient ^10^. However, these are usually conducted in high-income countries, whose dietary patterns differ from middle- and low-income countries, where evidence is limited.

Research on the content of pediatric supplements in relation to dietary recommendations is crucial, given the growing market and potential risks. This study aimed to describe the nutritional content reported in vitamin and mineral dietary supplements aimed at children on the Peruvian market and compare their contribution with daily intake recommendations.

**KEY MESSAGES**

Motivation for the study. Most vitamin and mineral supplements are presented in ways to appeal to children, such as gummies, and use marketing techniques aimed at them, which could influence the consumption and demand for these products.

Main findings. Several supplements exceeded the recommended intakes, with approximately half of the products containing vitamins C, D, A, and B6 exceeding the recommended dietary allowances (RDAs)/adequate intakes (AIs) for children aged 1 to 3 years.

Public health implications. Although no median vitamin and mineral doses exceeded the tolerable upper intake level (UL), a quarter of the products exceeded these limits for vitamin A and folate in children aged 1 to 3 years, highlighting the need for stricter regulation.

## THE STUDY

We conducted a cross-sectional observational study. One of the authors (LSG) selected the products between December 15 and 22, 2022, from the websites of nationally available drugstores. The “Dietary supplements for children” section was accessed, in which we identified 46 products described as vitamins and/or minerals (n=46). We included those with the following: (a) a statement of use for children or (b) the presence of marketing strategies aimed at children (promotional language, child-friendly typography, use of animated characters). The one with the highest net content was included in the case of products with different sizes.

We purchased 35 products due to the unavailability of some of them at the time of shipment. After verifying the name, brand, and description, one product described as a probiotic supplement was excluded.

The products were photographed to capture each side of the packaging, the contents of the insert, and any measuring instruments included. Based on the photos, the following information was registered in an Excel spreadsheet: presentation, country of origin, type (single nutrient or multi-micronutrient), marketing techniques, list of ingredients, nutritional composition (vitamin and/or mineral content), and stated recommendations (dosage, frequency, and age group).

Nutritional values were standardized according to the units used by the US Institute of Medicine (IOM) ^11^. We examined the content of vitamins A, D, E, C, thiamine, riboflavin, niacin, pantothenic acid, B6, biotin, folate, B12, and choline, and minerals calcium, zinc, iron, iodine, sodium, potassium, magnesium, manganese, copper, fluorine, phosphorus, and selenium. To ensure data quality, the information was entered twice by four researchers (AMG, AQM, HGDH, and RIBO). The records were compared to resolve discrepancies by reviewing photos by a fifth researcher (JGV).

The variable of interest was the nutrient content expressed as the proportion of the daily dose relative to the dietary reference intakes (DRI) for the following age groups: 1 to 3 years, 4 to 8 years, 9 to 13 years, and 14 to 18 years ^11^. This strategy was chosen due to the lack of uniformity in the declaration of nutritional content (e.g., vitamin C 45 mg/2 gummies; iron 25 mg/mL). The age groups were defined according to the IOM, which establishes different recommendations by age and, in some cases, by sex ^11^.

The IOM establishes the DRIs, which include the RDA or adequate intake (AI) when it was not possible to estimate the RDA, and the UL (Supplementary Table 1) ^11^. The percentage of nutrient content was calculated in relation to the RDA or AI and with respect to the UL. These values were obtained by dividing the daily dose of the nutrient by the corresponding DRI. The respective adequacy was calculated when the denominator was the RDA or AI; and the UL percentage was obtained when the denominator was the UL.

**Table 1:** Characteristics of the analyzed dietary supplements (n=34).

Characteristics		n	%
Product origin			
	National	16	47.1
	International	18	52.9
Presentation			
	Gummies	13	38.3
	Syrup	8	23.5
	Effervescent tablets	3	8.8
	Powder	3	8.8
	Other ^a^	7	20.6
Presence of octagon			
	Yes	5	14.7
	No	29	85.3
Health registration presence			
	Yes	33	97.1
	No	1	2.9
Requires reconstitution			
	Yes	6	17.7
	No	28	82.3
Declares list of ingredients			
	Yes	14	41.2
	No	20	58.8
Nutrient content of the product			
	Only one	8	23.5
	Two or more	26	76.5
Presents marketing techniques for children			
	Yes	24	70.6
	No	10	29.4
Declare at least one prescription			
	Yes	28	82.3
	No	6	17.7
Declare age group for prescription			
	Yes	24	70.6
	No	10	29.4

a Includes oral solution in drops, coated tablets, and chewable tablets.

The daily dose was estimated according to the prescription on the insert or packaging, multiplying the nutritional content by the number of recommended doses per day for each age group. The portion size stated in the packaging was used as the recommended daily dose for products without any prescription (n=6). The same indication was used for all groups in products with a single prescription for all age groups (n=1). These decisions were made based on the “reasonable consumer” principle proposed by the National Institute for the Defense of Competition and the Protection of Intellectual Property ^12^.

We used the highest value to estimate the highest possible consumption in the case of products with prescriptions expressed as ranges (e.g., “consume 1 to 3 tablets per day”). Finally, the adequacy of the RDA or AI and the percentage of the UL was calculated for each nutrient, age group, and product according to the following formulas:


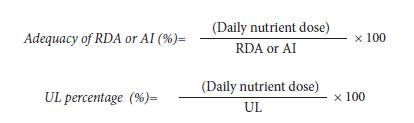


Because the IOM establishes sex-differentiated recommendations for some micro-nutrients in the 9- to 13- and 14- to 18-year-old age groups, calculations in these scenarios were also performed separately by sex.

These calculations were not performed for cobalt (n=1) due to the lack of DRI.

STATA version 17.0 was used for descriptive analysis, with absolute frequencies and percentages for categorical variables. RDA or AI adjustments and UL percentages were reported using the median, 25th and 75th percentiles, and minimum and maximum values, due to the high dispersion in the data ^5^, thus avoiding biases due to outliers. Box plots were constructed using SPSS version 27.0 to visualize RDA or AI and UL adequacy by age group, setting 100% as the contrast line on the Y axis.

The study was based on information declared on the supplement packaging, so it did not pose a risk to humans and did not require approval from an institutional ethics committee.

## FINDINGS

Thirty-four products were analyzed. The most common nutrients were vitamin C (n=23), vitamin D (n=22), vitamin A (n=16), zinc (n=15), and vitamin B6 (n=14), while the least common were vitamin K, choline, copper, cobalt, fluoride, phosphorus, selenium, and molybdenum (Supplementary Table 2). Approximately half of the supplements were manufactured domestically, and 76.5% contained multiple micronutrients (≥2). The most common presentation was gummies, and 82.4% did not require reconstitution. It is noteworthy that 58.8% did not list ingredients and 70.6% used marketing techniques aimed at children. In addition, 17.7% did not indicate prescription and 29.4% did not specify doses according to age group (Table 1).

**Figure 1:**
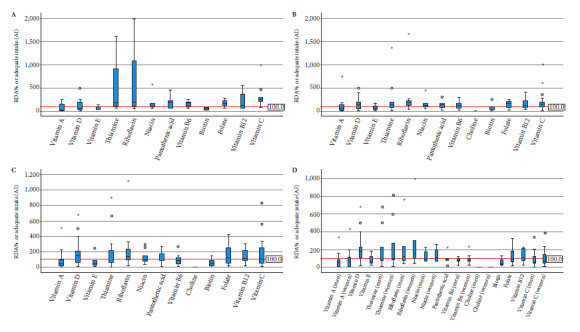
Percentage of dietary recommendations (RDA) or adequate intake (AI) of vitamins according to age group.

The percentage of adequacy of the RDA or AI revealed that, in children aged 1 to 3 years, the median dose of thiamine, riboflavin, vitamin B6, folate, B12, and vitamin C exceeded the recommendations. The same was observed for vitamin D, thiamine, riboflavin, niacin, pantothenic acid, vitamin B6, folate, vitamin B12, and vitamin C in the 4-8 age group. In the 9-13 and 14-18 age groups, the median values for vitamin D, riboflavin, and vitamin B12 exceeded the recommendations (Figure 1).

The number of supplements evaluated for each vitamin and age group varied according to availability. The percentage adequacy of the RDA or AI for vitamins was calculated separately for children aged 1 to 3 years (A), 4 to 8 years (B), 9 to 13 years (C), and 14 to 18 years (D). The horizontal red line represents 100% adequacy for the RDA or AI.

**Figure 2:**
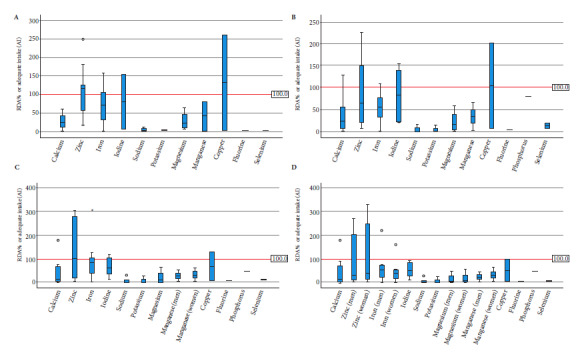
Percentage of recommended dietary allowances (RDA) or adequate intake (AI) of minerals according to age group.

Regarding minerals, the median zinc and copper doses exceeded the recommendations in the 1-3 age group, while in the 4-8 age group, only copper did so. In the 9-13 and 14-18 age groups, no median mineral intake exceeded the RDA or AI (Figure 2 and Supplementary Tables 3, 4, and 5).

The number of supplements evaluated for each mineral and age group varied according to availability. The percentage adequacy of the RDA or AI for vitamins was calculated separately for children aged 1 to 3 years (A), 4 to 8 years (B), 9 to 13 years (C), and 14 to 18 years (D). The horizontal red line represents 100% adequacy for the RDA or AI.

Although no median vitamin or mineral intake exceeded the UL, 25% of intakes exceeded the UL for vitamin A and folate in 1- to 3-year-olds and for folate in 4- to 13-year-olds (Supplementary Figures 1 and 2).

## DISCUSSION

Our results reveal important aspects about the vitamin and mineral content of dietary supplements for children on the Peruvian market. Multivitamin supplements were predominant and mainly presented as gummies, an attractive and easy-to-consume format for children. Among the analyzed nutrients, water-soluble vitamins such as the B complex and vitamin C, as well as fat-soluble vitamins A and D, were frequently present in doses that exceeded the RDA or AI. Among minerals, zinc stood out for exceeding the recommendations for children aged 1 to 3 years. Although the median nutrient doses did not exceed the UL, certain products exceeded these limits, especially for vitamin A and folate in the younger age groups.

These findings are consistent with studies conducted in other countries. In Canada, a study reported that most supplements for children contained nutrient doses higher than the daily recommendations ^5^. In the United States, nearly 70% of dietary supplements contained nutrient amounts equal to or higher than the RDAs ^13^. This situation may be driven by marketing strategies. In this study, most products used marketing aimed at children, which could influence the demand for and consumption of supplements, as documented in previous studies ^14^. This marketing strategy, coupled with easy-to-consume formats such as gummies and the high content of certain nutrients found in some products, may lead consumers to exceed nutrient recommendations. This is particularly concerning, considering that institutions such as the US Academy of Nutrition and Dietetics advise against the use of supplements in children with balanced diets that meet their requirements ^15^.

On the other hand, the use of multivitamin supplements, although it can facilitate the intake of multiple nutrients simultaneously, raises questions about possible interactions that affect the absorption and bioactivity of nutrients. For example, calcium and iron can interfere with the absorption of zinc and magnesium when included in the same formulation ^16^. This highlights the importance of evaluating the composition and combination of nutrients when developing and recommending supplements.

Another relevant finding were the supplements with doses exceeding the UL, as was the case of vitamin A. Vitamin A toxicity can cause serious adverse effects such as hepatotoxicity and visual disorders ^10^. Folate was also found in doses above the UL. Although studies have shown that, in children, intake may exceed this limit without identified adverse effects, high doses may mask vitamin B12 deficiencies and aggravate neuropathies ^17^. In the case of zinc, excess intake can cause nausea, vomiting, copper deficiencies, and growth problems ^18^, as well as an increased risk of anemia ^19^.

Our study has limitations. The review of nutrient content was based on information declared on packaging, without verification by laboratory analysis, which could introduce bias if the declared information was not accurate. In addition, the products were selected during a specific period in 2022 and from the “Dietary supplements for children” sections of online pharmacies, which may have excluded products intended for both adults and children available in other sections and may not represent the entire market. Finally, supplement consumption was not assessed, so it is not possible to confirm that the doses reflect actual consumption in the population. 

It is important to conduct further research analyzing the impact of supplement consumption on total nutrient intake in Peruvian children, as well as to study the interactions between nutrients in multivitamin supplements. At the same time, parents need to be educated about their consumption to ensure safety and efficacy. In this regard, it is essential to implement rigorous labeling, such as that established by the FDA ^20^, and to have statements from regulatory and health authorities, as established by the Academy of Nutrition and Dietetics in the United States ^15^.

In conclusion, the analysis of vitamin and mineral supplements for children on the Peruvian market showed variability in the adequacy of the doses. Several products exceed the recommended intakes and, in some cases, the maximum tolerable levels of vitamin A and folate. These findings highlight the need to monitor these products and ensure clear labeling.
