# Supplementary material for: Nutritional content of vitamin and mineral supplements aimed at children in the peruvian market: analysis of compliance with recommendations
Source: Rev Peru Med Exp Salud Publica. 2025 Mar 17;42(1):82–7. doi: 10.17843/rpmesp.2025.421.14256 (PMC12176023; doi:10.17843/rpmesp.2025.421.14256)
Supplement: Supplementary material. — Available in the electronic version of the RPMESP. [file rpmesp-42-01-14256-s001.docx]

Material suplementario

| **Tabla suplementaria 1. Ingestas dietéticas de referencia (DRI) de vitaminas y minerales por grupo etario** | | | | | | | |
| --- | --- | --- | --- | --- | --- | --- | --- |
| **Vitaminas (unidad)** | **DRI** | **Grupo de edad** | | | | | |
|  |  | **1 a 3 años** | **4 a 8 años** | **9 a 13 años (varón)** | **9 a 13 años (mujer)** | **14 a 18 años (varón)** | **14 a 18 años (mujer)** |
| Vitamina A (mcg) | RDA | 300 | 400 | 600 | 600 | 900 | 700 |
|  | UL | 600 | 900 | 1700 | 1700 | 2800 | 2800 |
| Vitamina D (mcg) | AI | 5 | 5 | 5 | 5 | 5 | 5 |
|  | UL | 50 | 50 | 50 | 50 | 50 | 50 |
| Vitamina E (mg) | RDA | 6 | 7 | 11 | 11 | 15 | 15 |
|  | UL | 200 | 300 | 600 | 600 | 800 | 800 |
| Vitamina K (mcg) | AI | 30 | 55 | 60 | 60 | 75 | 75 |
|  | UL | ND | ND | ND | ND | ND | ND |
| Tiamina (mg) | RDA | 0.5 | 0.6 | 0.9 | 0.9 | 1.2 | 1 |
|  | UL | ND | ND | ND | ND | ND | ND |
| Riboflavina (mg) | RDA | 0.5 | 0.6 | 0.9 | 0.9 | 1.3 | 1 |
|  | UL | ND | ND | ND | ND | ND | ND |
| Niacina (mg) | RDA | 6 | 8 | 12 | 12 | 16 | 14 |
|  | UL | 10 | 15 | 20 | 20 | 30 | 30 |
| Ácido pantoténico (mg) | AI | 2 | 3 | 4 | 4 | 5 | 5 |
|  | UL | ND | ND | ND | ND | ND | ND |
| Vitamina B6 (mg) | RDA | 0.5 | 0.6 | 1 | 1 | 1.3 | 1.2 |
|  | UL | 30 | 40 | 60 | 60 | 80 | 80 |
| Colina (mg) | AI | 200 | 250 | 375 | 375 | 550 | 400 |
|  | UL | 1000 | 1000 | 2000 | 2000 | 3000 | 3000 |
| Biotina (mg) | AI | 8 | 12 | 20 | 20 | 25 | 25 |
|  | UL | ND | ND | ND | ND | ND | ND |
| Vitamina B9 (mcg) | RDA | 150 | 200 | 300 | 300 | 400 | 400 |
|  | UL | 300 | 400 | 600 | 600 | 800 | 800 |
| Vitamina B12 (mcg) | RDA | 0.9 | 1.2 | 1.8 | 1.8 | 2.4 | 2.4 |
|  | UL | 300 | 400 | 600 | 600 | 800 | 800 |
| Vitamina C (mg) | RDA | 15 | 25 | 45 | 45 | 75 | 65 |
|  | UL | 400 | 650 | 1200 | 1200 | 1800 | 1800 |
|  |  |  |  |  |  |  |  |
| **Minerales (unidad)** | **DRI** | **Grupo de edad** | | | | | |
|  |  | **1 a 3 años** | **4 a 8 años** | **9 a 13 años (varón)** | **9 a 13 años (mujer)** | **14 a 18 años (varón)** | **14 a 18 años (mujer)** |
| Calcio (mg) | AI | 500 | 800 | 1300 | 1300 | 1300 | 1300 |
|  | UL | 2500 | 2500 | 2500 | 2500 | 2500 | 2500 |
| Zinc (mg) | RDA | 3 | 5 | 8 | 8 | 11 | 9 |
|  | UL | 7 | 12 | 23 | 23 | 34 | 34 |
| Hierro (mg) | RDA | 7 | 10 | 8 | 8 | 11 | 15 |
|  | UL | 40 | 40 | 40 | 40 | 45 | 45 |
| Yodo (mcg) | RDA | 90 | 90 | 120 | 120 | 150 | 150 |
|  | UL | 200 | 300 | 600 | 600 | 900 | 900 |
| Sodio (g) | AI | 1 | 1.2 | 1.5 | 1.5 | 1.5 | 1.5 |
|  | UL | 1.5 | 1.9 | 2.2 | 2.2 | 2.3 | 2.3 |
| Potasio (g) | AI | 3 | 3.8 | 4.5 | 4.5 | 4.7 | 4.7 |
|  | UL | ND | ND | ND | ND | ND | ND |
| Magnesio (mg) | RDA | 80 | 130 | 240 | 240 | 410 | 360 |
|  | UL | 65 | 110 | 350 | 350 | 350 | 350 |
| Manganeso (mg) | AI | 1.2 | 1.5 | 1.9 | 1.6 | 2.2 | 1.6 |
|  | UL | 2 | 3 | 6 | 6 | 9 | 9 |
| Cobre (mcg) | RDA | 340 | 440 | 700 | 700 | 890 | 890 |
|  | UL | 1000 | 3000 | 5000 | 5000 | 8000 | 8000 |
| Cobalto | ND | ND | ND | ND | ND | ND | ND |
|  | UL | ND | ND | ND | ND | ND | ND |
| Flúor (mg) | AI | 0.7 | 1 | 2 | 2 | 3 | 3 |
|  | UL | 1.3 | 2.2 | 10 | 10 | 10 | 10 |
| Fósforo (mg) | RDA | 460 | 500 | 1250 | 1250 | 1250 | 1250 |
|  | UL | 3000 | 3000 | 4000 | 4000 | 4000 | 4000 |
| Selenio (mcg) | RDA | 20 | 30 | 40 | 40 | 55 | 55 |
|  | UL | 90 | 150 | 280 | 280 | 400 | 400 |
| Molibdeno (mcg) | RDA | 17 | 22 | 34 | 34 | 43 | 43 |
|  | UL | 300 | 600 | 1100 | 1100 | 1700 | 1700 |
| RDA: recomendaciones diarias de ingesta. AI: ingesta adecuada. UL: dosis de ingesta máxima tolerable. Cuando no existe un RDA definido para un nutriente se utiliza el valor del AI | | | | | | | |

| **Tabla suplementaria 2. Frecuencia de presencia de vitaminas y minerales en los productos analizados (n = 34)** | | | | | | |
| --- | --- | --- | --- | --- | --- | --- |
| ***Vitaminas*** | **n** | **%** |  | ***Minerales*** | **n** | **%** |
| **Vitamina A** |  |  |  | **Calcio** |  |  |
| Si | 16 | 47.1 |  | Si | 13 | 38.2 |
| No | 18 | 52.9 |  | No | 21 | 61.8 |
| **Vitamina D** |  |  |  | **Zinc** |  |  |
| Si | 22 | 64.7 |  | Si | 15 | 44.1 |
| No | 12 | 35.3 |  | No | 19 | 55.9 |
| **Vitamina E** |  |  |  | **Hierro** |  |  |
| Si | 11 | 32.4 |  | Si | 11 | 32.4 |
| No | 23 | 67.7 |  | No | 23 | 67.7 |
| **Vitamina K** |  |  |  | **Yodo** |  |  |
| Si | 0 | 0 |  | Si | 5 | 14.7 |
| No | 34 | 100 |  | No | 29 | 85.3 |
| **Tiamina** |  |  |  | **Sodio** |  |  |
| Si | 12 | 35.3 |  | Si | 7 | 20.6 |
| No | 22 | 64.7 |  | No | 27 | 79.4 |
| **Riboflavina** |  |  |  | **Potasio** |  |  |
| Si | 12 | 35.3 |  | Si | 4 | 11.8 |
| No | 22 | 64.7 |  | No | 30 | 88.2 |
| **Niacina** |  |  |  | **Magnesio** |  |  |
| Si | 11 | 32.4 |  | Si | 8 | 23.5 |
| No | 23 | 67.7 |  | No | 26 | 76.5 |
| **Ácido Pantoténico** |  |  |  | **Manganeso** |  |  |
| Si | 12 | 35.3 |  | Si | 3 | 8.8 |
| No | 22 | 64.7 |  | No | 31 | 91.2 |
| **Vitamina B6** |  |  |  | **Cobre** |  |  |
| Si | 14 | 41.2 |  | Si | 2 | 5.9 |
| No | 20 | 58.8 |  | No | 32 | 94.1 |
| **Colina** |  |  |  | **Cobalto** |  |  |
| Si | 2 | 5.9 |  | Si | 1 | 2.9 |
| No | 32 | 94.1 |  | No | 33 | 97.1 |
| **Biotina** |  |  |  | **Flúor** |  |  |
| Si | 5 | 14.7 |  | Si | 1 | 2.9 |
| No | 29 | 85.3 |  | No | 33 | 97.1 |
| **Folato** |  |  |  | **Fósforo** |  |  |
| Si | 10 | 29.4 |  | Si | 1 | 2.9 |
| No | 24 | 70.6 |  | No | 33 | 97.1 |
| **Vitamina B12** |  |  |  | **Selenio** |  |  |
| Si | 11 | 32.4 |  | Si | 2 | 5.9 |
| No | 23 | 67.7 |  | No | 32 | 94.1 |
| **Vitamina C** |  |  |  | **Molibdeno** |  |  |
| Si | 23 | 67.7 |  | Si | 0 | 0 |
| No | 11 | 32.4 |  | No | 34 | 100 |

| **Tabla suplementaria 3. Distribución de adecuación de nutrientes al RDA o AI, en los grupos de edad de 1 a 3 años y 4 a 8 años** | | | | | | | | | |
| --- | --- | --- | --- | --- | --- | --- | --- | --- | --- |
| **Vitaminas y minerales** |  | **1 a 3 años** | | |  |  | **4 a 8 años** | | |
|  | **n** | **Mediana** | **[p25; p75]** | **[Min; Max]** |  | **n** | **Mediana** | **[p25; p75]** | **[Min; Max]** |
| **Vitaminas** |  |  |  |  |  |  |  |  |  |
| Vitamina A | 6 | 25 | [5; 166.7] | [0; 249.9] |  | 15 | 62.5 | [9; 150] | [0; 750] |
| Vitamina D | 12 | 62.5 | [37.5; 200] | [13; 500] |  | 21 | 140 | [50; 200] | [13; 500] |
| Vitamina E | 3 | 50 | [50; 133.3] | [50; 133.3] |  | 10 | 71.4 | [38.9; 114.3] | [17; 171.4] |
| Tiamina | 4 | 180 | [110; 913] | [60; 1626] |  | 11 | 138.3 | [50; 200] | [2.2; 1355] |
| Riboflavina | 4 | 190 | [120; 1100] | [60; 2000] |  | 11 | 200 | [100; 250] | [30; 1666.7] |
| Niacina | 5 | 166.7 | [100; 166.7] | [56.7; 583.3] |  | 10 | 125 | [104.1; 168.8] | [50; 437.5] |
| Ácido Pantoténico | 5 | 200 | [60; 230] | [60; 460] |  | 11 | 133.3 | [80; 166.7] | [15.3; 306.7] |
| Vitamina B6 | 5 | 180 | [100; 200] | [60; 246] |  | 13 | 125 | [68.3; 166.7] | [25; 300] |
| Colina | 0 | - | - | - |  | 2 | 1.3 | [0.8; 1.7] | [0.8; 1.7] |
| Biotina | 2 | 44.8 | [14.6; 75] | [14.6; 75] |  | 5 | 38.9 | [33.3; 100] | [7; 250] |
| Folato | 4 | 166.8 | [116.7; 222.7] | [66.7; 278.3] |  | 10 | 162.5 | [83.5; 210] | [25; 255] |
| Vitamina B12 | 4 | 116.7 | [66.7; 361.1] | [66.7; 555.6] |  | 11 | 125 | [69.4; 250] | [37.5; 416.7] |
| Vitamina C | 11 | 250 | [166.7; 300] | [83.3; 1000] |  | 22 | 155 | [99.9; 200] | [6; 1000] |
| **Minerales** |  |  |  |  |  |  |  |  |  |
| Calcio | 8 | 25 | [11.5; 40.8] | [0; 60] |  | 13 | 23.9 | [6.1; 56.3] | [0; 128.9] |
| Zinc | 10 | 116.7 | [55; 125] | [16.7; 250] |  | 14 | 65 | [20; 150] | [6.3; 225] |
| Hierro | 7 | 71.4 | [23.6; 114.3] | [0; 157.1] |  | 11 | 56 | [30; 80] | [0; 108] |
| Yodo | 2 | 79.8 | [5.1; 154.4] | [5.1; 154.4] |  | 5 | 83.5 | [20.6; 138.9] | [19.1; 154.4] |
| Sodio | 4 | 2.4 | [0.04; 7.59] | [0.04; 10.5] |  | 7 | 0.6 | [0; 8.8] | [0; 15.6] |
| Potasio | 2 | 2.3 | [0.2; 4.4] | [0.2; 4.4] |  | 4 | 0.2 | [0.1; 7.2] | [0; 14] |
| Magnesio | 4 | 21.4 | [8.3; 46.9] | [4.9; 62.5] |  | 8 | 15.9 | [2.8; 38.5] | [1.2; 57.7] |
| Manganeso | 2 | 41 | [0.7; 81.3] | [0.7; 81.3] |  | 3 | 33.4 | [2.2; 65] | [2.2; 65] |
| Cobre | 2 | 131.9 | [1.9; 262] | [1.9; 262] |  | 2 | 104.1 | [5.8; 202.4] | [5.8; 202.4] |
| Flúor | 1 | 1.6 | * | * |  | 1 | 4.4 | * | * |
| Fósforo | 0 | - | - | - |  | 1 | 80 | * | * |
| Selenio | 1 | 2.1 | * | * |  | 2 | 12.78 | [5.56; 20] | [5.6; 20] |
| El tamaño de muestra (n) para el cálculo de la adecuación varió entre nutrientes, ya que no todos los productos contenían todos los nutrientes ni tenían prescripción para todos los grupos etarios . En el grupo de 1 a 3 años, no se pudo calcular la adecuación para colina ni fósforo, ya que, aunque dos productos contenían colina y uno fósforo, ninguno especificaba una prescripción para este grupo. Las celdas con asteriscos indican que no se calculó la medida correspondiente en la columna, debido a que el nutriente estuvo presente en un solo producto | | | | | | | | | |
|  | | | | | | | | | |
|  |  |  |  |  |  |  |  |  |  |
|  |  |  |  |  |  |  |  |  |  |

| **Tabla suplementaria 4. Distribución de adecuación de nutrientes al RDA o AI, en el grupo de edad de 9 a 13 años** | | | | |
| --- | --- | --- | --- | --- |
| **Vitaminas y minerales** | **n** | **Mediana** | **[p25; p75]** | **[Min; Max]** |
| **Vitaminas** |  |  |  |  |
| Vitamina A | 15 | 41.7 | [9; 125] | [0; 500] |
| Vitamina D | 20 | 150 | [59.3; 212.5] | [13; 675] |
| Vitamina E | 10 | 45.5 | [24.7; 87.3] | [10.8; 245.5] |
| Tiamina | 11 | 100 | [33.3; 300] | [1.5; 903.3] |
| Riboflavina | 11 | 133.3 | [100; 300] | [20; 1111.1] |
| Niacina | 10 | 97.9 | [83.3; 150] | [33.3; 291.7] |
| Ácido Pantoténico | 11 | 100 | [87.5; 230] | [11.5; 270] |
| Vitamina B6 | 13 | 86.1 | [41; 123] | [15; 270] |
| Colina | 2 | 0.9 | [0.6; 1.2] | [0.6; 1.2] |
| Biotina | 5 | 52.5 | [20; 90] | [4.2; 150] |
| Folato | 10 | 119.6 | [55.7; 250.5] | [16.7; 420] |
| Vitamina B12 | 11 | 111.1 | [83.3; 277.8] | [25; 300] |
| Vitamina C | 21 | 100 | [55.6; 250] | [6.7; 833.3] |
| **Minerales** |  |  |  |  |
| Calcio | 13 | 14.7 | [3.7; 69.2] | [0; 178.5] |
| Zinc | 14 | 40.6 | [18.8; 281.3] | [4; 303.8] |
| Hierro | 11 | 85 | [37.5; 105] | [0; 303.8] |
| Yodo | 5 | 62.6 | [34.7; 104.2] | [14.3; 115.8] |
| Sodio | 7 | 0.5 | [0; 9.6] | [0; 28] |
| Potasio | 4 | 0.3 | [0.1; 13.5] | [0; 26.6] |
| Magnesio | 8 | 11.8 | [1.5; 38.2] | [0.6; 62.5] |
| Manganeso (varón) | 3 | 26.3 | [4; 51.3] | [4; 51.3] |
| Manganeso (mujer) | 3 | 31.3 | [4.7; 60.9] | [4.7; 60.9] |
| Cobre | 2 | 31.3 | [8.1; 127.2] | [8.1; 127.2] |
| Flúor | 1 | 67.7 | * | * |
| Fósforo | 1 | 48 | * | * |
| Selenio | 2 | 12.2 | [9.4; 15] | [9.4; 15] |
| El tamaño de muestra (n) para el cálculo de la adecuación varió entre nutrientes, ya que no todos los productos contenían todos los nutrientes ni tenían prescripción para todos los grupos etarios. Las celdas con asteriscos indican que no se calculó la medida indicada en la columna debido a que el nutriente estuvo presente en solo un producto | | | | |

| **Tabla suplementaria 5. Distribución de adecuación de nutrientes al RDA o AI, en el grupo de edad de 14 a18 años** | | | | |
| --- | --- | --- | --- | --- |
| **Vitaminas y minerales** | **n** | **Mediana** | **[p25; p75]** | **[Min; Max]** |
| **Vitaminas** |  |  |  |  |
| Vitamina A (varón) | 10 | 49.3 | [1.7; 83.3] | [0; 333.3] |
| Vitamina A (mujer) | 10 | 63.3 | [2.1; 107.1] | [0; 428.6] |
| Vitamina D | 18 | 100 | [100; 225] | [13; 675] |
| Vitamina E | 7 |  | [33; 180] | [18.1; 180] |
| Tiamina (varón) | 10 | 79.2 | [66.7; 225] | [3.3; 677.5] |
| Tiamina (mujer) | 10 | 95 | [80; 270] | [3.9; 813] |
| Riboflavina (varón) | 10 | 118.8 | [69.2; 230.8] | [32.7; 769.2] |
| Riboflavina (mujer) | 10 | 142.3 | [90; 300] | [42.5; 1000] |
| Niacina (varón) | 8 | 89.1 | [62.5; 173.7] | [62.5; 218.8] |
| Niacina (mujer) | 8 | 101.8 | [71.4; 198.5] | [71.4; 250] |
| Ácido Pantoténico | 8 | 85.9 | [71; 96] | [9.2; 216] |
| Vitamina B6 (varón) | 10 | 73.1 | [63.1; 94.6] | [15.8; 207.7] |
| Vitamina B6 (mujer) | 10 | 79.2 | [68.3; 102.5] | [17.1; 225] |
| Colina (varón) | 2 | 0.6 | [0.4; 0.8] | [0.4; 0.8] |
| Colina (mujer) | 2 | 0.8 | [0.5; 1.1] | [0.5; 1.1] |
| Biotina | 3 | 42 | [16; 120] | [16; 120] |
| Folato | 8 | 89.7 | [52.1; 176.3] | [12.5; 315] |
| Vitamina B12 | 9 | 104.1 | [75; 208.3] | [18.8; 225] |
| Vitamina C (varón) | 16 | 53.3 | [41.7; 121.7] | [4; 333.3] |
| Vitamina C (mujer) | 16 | 61.5 | [48.1; 140.4] | [4.6; 384.6] |
| **Minerales** |  |  |  |  |
| Calcio | 12 | 13.5 | [2.4; 72.1] | [0; 178.5] |
| Zinc (varón) | 12 | 31.8 | [11.5; 204.5] | [2.9; 272.7] |
| Zinc (mujer) | 12 | 38.9 | [14.1; 250] | [3.5; 333.3] |
| Hierro (varón) | 8 | 53.6 | [22.2; 74.5] | [0; 220.9] |
| Hierro (mujer) | 8 | 39.3 | [16.3; 54.7] | [0; 162] |
| Yodo | 5 | 50.1 | [27.8; 83.3] | [11.5; 92.7] |
| Sodio | 6 | 3.8 | [0; 9.6] | [0; 28] |
| Potasio | 4 | 0.3 | [0.1; 12.9] | [0; 25.5] |
| Magnesio (varón) | 8 | 6.9 | [0.9; 28.5] | [0.4; 48.8] |
| Magnesio (mujer) | 8 | 7.8 | [1; 32.4] | [0.4; 55.6] |
| Manganeso (varón) | 3 | 22.8 | [3.4; 44.3] | [3.4; 44.3] |
| Manganeso (mujer) | 3 | 31.3 | [4.7; 60.9] | [4.7; 60.9] |
| Cobre | 2 | 53.2 | [6.4; 100.1] | [6.4; 100.1] |
| Flúor | 1 | 3.3 | * | * |
| Fósforo | 1 | 48 | * | * |
| Selenio | 1 | 6.8 | * | * |
| El tamaño de muestra (n) para el cálculo de la adecuación varió entre nutrientes, ya que no todos los productos contenían todos los nutrientes ni tenían prescripción para todos los grupos etarios. Las celdas con asteriscos indican que no se calculó la medida indicada en la columna debido a que el nutriente estuvo presente en solo un producto | | | | |

| **Tabla suplementaria 6. Proporción de productos cuya composición de vitaminas supera las recomendaciones de ingesta diaria (RDA o AI y UL) por grupo etario** | | | | | | | |
| --- | --- | --- | --- | --- | --- | --- | --- |
| **Nutriente evaluado** | **Grupo etario** | **Total de productos (n)** | **Adecuación (%)** | **RDA/AI** | | **UL** | |
|  |  |  |  |  |  |  |  |
|  |  |  |  | **n** | **%** | **n** | **%** |
| **Vitamina A** | 1 a 3 años | 6 | ≤100% | 4 | 66.67 | 4 | 66.67 |
|  |  |  | >100% | 2 | 33.33 | 2 | 33.33 |
|  | 4 a 8 años | 15 | ≤100% | 9 | 60.00 | 13 | 86.67 |
|  |  |  | >100% | 6 | 40.00 | 2 | 13.33 |
|  | 9 a 13 años | 15 | ≤100% | 11 | 73.33 | 14 | 93.33 |
|  |  |  | >100% | 4 | 26.67 | 1 | 6.67 |
|  | 14 a 18 años (varón) | 10 | ≤100% | 8 | 80.00 | 9 | 90.00 |
|  |  |  | >100% | 2 | 20.00 | 1 | 10.00 |
|  | 14 a 18 años (mujer) | 10 | ≤100% | 7 | 70.00 | 9 | 90.00 |
|  |  |  | >100% | 3 | 30.00 | 1 | 10.00 |
| **Vitamina D** | 1 a 3 años | 12 | ≤100% | 7 | 58.33 | 12 | 100.00 |
|  |  |  | >100% | 5 | 41.67 | 0 | 0.00 |
|  | 4 a 8 años | 21 | ≤100% | 10 | 47.62 | 21 | 100.00 |
|  |  |  | >100% | 11 | 52.38 | 0 | 0.00 |
|  | 9 a 13 años | 20 | ≤100% | 7 | 35.00 | 20 | 100.00 |
|  |  |  | >100% | 13 | 65.00 | 0 | 0.00 |
|  | 14 a 18 años | 18 | ≤100% | 5 | 27.78 | 18 | 100.00 |
|  |  |  | >100% | 13 | 72.22 | 0 | 0.00 |
| **Vitamina E** | 1 a 3 años | 3 | ≤100% | 2 | 66.67 | 3 | 100.00 |
|  |  |  | >100% | 1 | 33.33 | 0 | 0.00 |
|  | 4 a 8 años | 10 | ≤100% | 7 | 70.00 | 10 | 100.00 |
|  |  |  | >100% | 3 | 30.00 | 0 | 0.00 |
|  | 9 a 13 años | 10 | ≤100% | 9 | 90.00 | 10 | 100.00 |
|  |  |  | >100% | 1 | 10.00 | 0 | 0.00 |
|  | 14 a 18 años | 7 | ≤100% | 5 | 71.43 | 7 | 100.00 |
|  |  |  | >100% | 2 | 28.57 | 0 | 0.00 |
| **Tiamina** | 1 a 3 años | 4 | ≤100% | 1 | 25.00 |  |  |
|  |  |  | >100% | 3 | 75.00 |  |  |
|  | 4 a 8 años | 11 | ≤100% | 2 | 18.18 |  |  |
|  |  |  | >100% | 9 | 81.82 |  |  |
|  | 9 a 13 años | 11 | ≤100% | 6 | 54.55 |  |  |
|  |  |  | >100% | 5 | 45.45 |  |  |
|  | 14 a 18 años (varón) | 10 | ≤100% | 7 | 70.00 |  |  |
|  |  |  | >100% | 3 | 30.00 |  |  |
|  | 14 a 18 años (mujer) | 10 | ≤100% | 6 | 60.00 |  |  |
|  |  |  | >100% | 4 | 40.00 |  |  |
| **Riboflavina** | 1 a 3 años | 4 | ≤100% | 1 | 25.00 |  |  |
|  |  |  | >100% | 3 | 75.00 |  |  |
|  | 4 a 8 años | 11 | ≤100% | 2 | 18.18 |  |  |
|  |  |  | >100% | 9 | 81.82 |  |  |
|  | 9 a 13 años | 11 | ≤100% | 4 | 36.36 |  |  |
|  |  |  | >100% | 7 | 63.64 |  |  |
|  | 14 a 18 años (varón) | 10 | ≤100% | 4 | 40.00 |  |  |
|  |  |  | >100% | 6 | 60.00 |  |  |
|  | 14 a 18 años (mujer) | 10 | ≤100% | 4 | 40.00 |  |  |
|  |  |  | >100% | 6 | 60.00 |  |  |
| **Niacina** | 1 a 3 años | 5 | ≤100% | 2 | 40.00 | 4 | 80.00 |
|  |  |  | >100% | 3 | 60.00 | 1 | 20.00 |
|  | 4 a 8 años | 10 | ≤100% | 2 | 20.00 | 9 | 90.00 |
|  |  |  | >100% | 8 | 80.00 | 1 | 10.00 |
|  | 9 a 13 años | 10 | ≤100% | 5 | 50.00 | 8 | 80.00 |
|  |  |  | >100% | 5 | 50.00 | 2 | 20.00 |
|  | 14 a 18 años (varón) | 8 | ≤100% | 5 | 62.50 | 6 | 75.00 |
|  |  |  | >100% | 3 | 37.50 | 2 | 25.00 |
|  | 14 a 18 años (mujer) | 8 | ≤100% | 4 | 50.00 | 6 | 75.00 |
|  |  |  | >100% | 4 | 50.00 | 2 | 25.00 |
| **Ácido Pantoténico** | 1 a 3 años | 5 | ≤100% | 2 | 40.00 |  |  |
|  |  |  | >100% | 3 | 60.00 |  |  |
|  | 4 a 8 años | 11 | ≤100% | 4 | 36.36 |  |  |
|  |  |  | >100% | 7 | 63.64 |  |  |
|  | 9 a 13 años | 11 | ≤100% | 6 | 54.55 |  |  |
|  |  |  | >100% | 5 | 45.45 |  |  |
|  | 14 a 18 años | 8 | ≤100% | 7 | 87.50 |  |  |
|  |  |  | >100% | 1 | 12.50 |  |  |
| **Vitamina B6** | 1 a 3 años | 5 | ≤100% | 2 | 40.00 | 5 | 100.00 |
|  |  |  | >100% | 3 | 60.00 | 0 | 0.00 |
|  | 4 a 8 años | 13 | ≤100% | 5 | 38.46 | 13 | 100.00 |
|  |  |  | >100% | 8 | 61.54 | 0 | 0.00 |
|  | 9 a 13 años | 13 | ≤100% | 9 | 69.23 | 13 | 100.00 |
|  |  |  | >100% | 4 | 30.77 | 0 | 0.00 |
|  | 14 a 18 años (varón) | 10 | ≤100% | 8 | 80.00 | 10 | 100.00 |
|  |  |  | >100% | 2 | 20.00 | 0 | 0.00 |
|  | 14 a 18 años (mujer) | 10 | ≤100% | 6 | 60.00 | 10 | 100.00 |
|  |  |  | >100% | 4 | 40.00 | 0 | 0.00 |
| **Colina** | 1 a 3 años | 0 | ≤100% | - | - | - | - |
|  |  |  | >100% | - | - | - | - |
|  | 4 a 8 años | 2 | ≤100% | 2 | 100.00 | 2 | 100.00 |
|  |  |  | >100% | 0 | 0.00 | 0 | 0.00 |
|  | 9 a 13 años | 2 | ≤100% | 2 | 100.00 | 2 | 100.00 |
|  |  |  | >100% | 0 | 0.00 | 0 | 0.00 |
|  | 14 a 18 años (varón) | 2 | ≤100% | 2 | 100.00 | 2 | 100.00 |
|  |  |  | >100% | 0 | 0.00 | 0 | 0.00 |
|  | 14 a 18 años (mujer) | 2 | ≤100% | 2 | 100.00 | 2 | 100.00 |
|  |  |  | >100% | 0 | 0.00 | 0 | 0.00 |
| **Biotina** | 1 a 3 años | 2 | ≤100% | 2 | 100.00 |  |  |
|  |  |  | >100% | 0 | 0.00 |  |  |
|  | 4 a 8 años | 5 | ≤100% | 4 | 80.00 |  |  |
|  |  |  | >100% | 1 | 20.00 |  |  |
|  | 9 a 13 años | 5 | ≤100% | 4 | 80.00 |  |  |
|  |  |  | >100% | 1 | 20.00 |  |  |
|  | 14 a 18 años | 3 | ≤100% | 2 | 66.67 |  |  |
|  |  |  | >100% | 1 | 33.33 |  |  |
| **Folato** | 1 a 3 años | 4 | ≤100% | 1 | 25.00 | 3 | 75.00 |
|  |  |  | >100% | 3 | 75.00 | 1 | 25.00 |
|  | 4 a 8 años | 10 | ≤100% | 4 | 40.00 | 6 | 60.00 |
|  |  |  | >100% | 6 | 60.00 | 4 | 40.00 |
|  | 9 a 13 años | 10 | ≤100% | 5 | 50.00 | 7 | 70.00 |
|  |  |  | >100% | 5 | 50.00 | 3 | 30.00 |
|  | 14 a 18 años | 8 | ≤100% | 4 | 50.00 | 6 | 75.00 |
|  |  |  | >100% | 4 | 50.00 | 2 | 25.00 |
| **Vitamina B12** | 1 a 3 años | 4 | ≤100% | 2 | 50.00 |  |  |
|  |  |  | >100% | 2 | 50.00 |  |  |
|  | 4 a 8 años | 11 | ≤100% | 4 | 36.36 |  |  |
|  |  |  | >100% | 7 | 63.64 |  |  |
|  | 9 a 13 años | 11 | ≤100% | 5 | 45.45 |  |  |
|  |  |  | >100% | 6 | 54.55 |  |  |
|  | 14 a 18 años | 9 | ≤100% | 4 | 44.44 |  |  |
|  |  |  | >100% | 5 | 55.56 |  |  |
| **Vitamina C** | 1 a 3 años | 11 | ≤100% | 1 | 9.09 | 11 | 100.00 |
|  |  |  | >100% | 10 | 90.91 | 0 | 0.00 |
|  | 4 a 8 años | 22 | ≤100% | 9 | 40.91 | 22 | 100.00 |
|  |  |  | >100% | 13 | 59.09 | 0 | 0.00 |
|  | 9 a 13 años | 21 | ≤100% | 11 | 52.38 | 21 | 100.00 |
|  |  |  | >100% | 10 | 47.62 | 0 | 0.00 |
|  | 14 a 18 años (varón) | 16 | ≤100% | 12 | 75.00 | 16 | 100.00 |
|  |  |  | >100% | 4 | 25.00 | 0 | 0.00 |
|  | 14 a 18 años (mujer) | 16 | ≤100% | 11 | 68.75 | 16 | 100.00 |
|  |  |  | >100% | 5 | 31.25 | 0 | 0.00 |
| Las celdas en gris representan los nutrientes para los cuales no existe UL reportado | | | | | | | |

| **Tabla suplementaria 7. Proporción de productos cuya composición de minerales supera las recomendaciones de ingesta diaria (RDA o AI y UL) por grupo etario** | | | | | | | |  |
| --- | --- | --- | --- | --- | --- | --- | --- | --- |
| **Nutriente evaluado** | **Grupo etario** | **Total de productos (n)** | **Adecuación (%)** | **RDA/AI** | | **UL** | |  |
|  |  |  |  |  |  |  |  |  |
|  |  |  |  | **n** | **%** | **n** | **%** |  |
| **Calcio** | 1 a 3 años | 8 | ≤100% | 8 | 100 | 8 | 100 |  |
|  |  |  | >100% | 0 | 0 | 0 | 0 |  |
|  | 4 a 8 años | 13 | ≤100% | 12 | 92.31 | 13 | 100 |  |
|  |  |  | >100% | 1 | 7.69 | 0 | 0 |  |
|  | 9 a 13 años | 13 | ≤100% | 12 | 92.31 | 13 | 100 |  |
|  |  |  | >100% | 1 | 7.69 | 0 | 0 |  |
|  | 14 a 18 años | 12 | ≤100% | 11 | 91.67 | 12 | 100 |  |
|  |  |  | >100% | 1 | 8.33 | 0 | 0 |  |
| **Zinc** | 1 a 3 años | 10 | ≤100% | 3 | 30 | 9 | 90 |  |
|  |  |  | >100% | 7 | 70 | 1 | 10 |  |
|  | 4 a 8 años | 14 | ≤100% | 10 | 71.43 | 14 | 100 |  |
|  |  |  | >100% | 4 | 28.57 | 0 | 0 |  |
|  | 9 a 13 años | 14 | ≤100% | 9 | 64.29 | 13 | 92.86 |  |
|  |  |  | >100% | 5 | 35.71 | 1 | 7.14 |  |
|  | 14 a 18 años (varón) | 12 | ≤100% | 7 | 58.33 | 12 | 100 |  |
|  |  |  | >100% | 5 | 41.67 | 0 | 0 |  |
|  | 14 a 18 años (mujer) | 12 | ≤100% | 7 | 58.33 | 12 | 100 |  |
|  |  |  | >100% | 5 | 41.67 | 0 | 0 |  |
| **Hierro** | 1 a 3 años | 7 | ≤100% | 5 | 71.43 | 7 | 100 |  |
|  |  |  | >100% | 2 | 28.57 | 0 | 0 |  |
|  | 4 a 8 años | 11 | ≤100% | 10 | 90.91 | 11 | 100 |  |
|  |  |  | >100% | 1 | 9.09 | 0 | 0 |  |
|  | 9 a 13 años | 11 | ≤100% | 8 | 72.73 | 11 | 100 |  |
|  |  |  | >100% | 3 | 27.27 | 0 | 0 |  |
|  | 14 a 18 años (varón) | 8 | ≤100% | 7 | 87.5 | 8 | 100 |  |
|  |  |  | >100% | 1 | 12.5 | 0 | 0 |  |
|  | 14 a 18 años (mujer) | 8 | ≤100% | 7 | 87.5 | 8 | 100 |  |
|  |  |  | >100% | 1 | 12.5 | 0 | 0 |  |
| **Yodo** | 1 a 3 años | 2 | ≤100% | 1 | 50 | 2 | 100 |  |
|  |  |  | >100% | 1 | 50 | 0 | 0 |  |
|  | 4 a 8 años | 5 | ≤100% | 3 | 60 | 5 | 100 |  |
|  |  |  | >100% | 2 | 40 | 0 | 0 |  |
|  | 9 a 13 años | 5 | ≤100% | 3 | 60 | 5 | 100 |  |
|  |  |  | >100% | 2 | 40 | 0 | 0 |  |
|  | 14 a 18 años | 5 | ≤100% | 5 | 100 | 5 | 100 |  |
|  |  |  | >100% | 0 | 0 | 0 | 0 |  |
| **Sodio** | 1 a 3 años | 4 | ≤100% | 4 | 100 | 4 | 100 |  |
|  |  |  | >100% | 0 | 0 | 0 | 0 |  |
|  | 4 a 8 años | 7 | ≤100% | 7 | 100 | 7 | 100 |  |
|  |  |  | >100% | 0 | 0 | 0 | 0 |  |
|  | 9 a 13 años | 7 | ≤100% | 7 | 100 | 7 | 100 |  |
|  |  |  | >100% | 0 | 0 | 0 | 0 |  |
|  | 14 a 18 años | 6 | ≤100% | 6 | 100 | 6 | 100 |  |
|  |  |  | >100% | 0 | 0 | 0 | 0 |  |
| **Potasio** | 1 a 3 años | 2 | ≤100% | 2 | 100 |  |  |  |
|  |  |  | >100% | 0 | 0 |  |  |  |
|  | 4 a 8 años | 4 | ≤100% | 4 | 100 |  |  |  |
|  |  |  | >100% | 0 | 0 |  |  |  |
|  | 9 a 13 años | 4 | ≤100% | 4 | 100 |  |  |  |
|  |  |  | >100% | 0 | 0 |  |  |  |
|  | 14 a 18 años | 4 | ≤100% | 4 | 100 |  |  |  |
|  |  |  | >100% | 0 | 0 |  |  |  |
| **Magnesio** | 1 a 3 años | 4 | ≤100% | 4 | 100 | 4 | 100 |  |
|  |  |  | >100% | 0 | 0 | 0 | 0 |  |
|  | 4 a 8 años | 8 | ≤100% | 8 | 100 | 8 | 100 |  |
|  |  |  | >100% | 0 | 0 | 0 | 0 |  |
|  | 9 a 13 años | 8 | ≤100% | 8 | 100 | 8 | 100 |  |
|  |  |  | >100% | 0 | 0 | 0 | 0 |  |
|  | 14 a 18 años (varón) | 8 | ≤100% | 8 | 100 | 8 | 100 |  |
|  |  |  | >100% | 0 | 0 | 0 | 0 |  |
|  | 14 a 18 años (mujer) | 8 | ≤100% | 8 | 100 | 8 | 100 |  |
|  |  |  | >100% | 0 | 0 | 0 | 0 |  |
| **Manganeso** | 1 a 3 años | 2 | ≤100% | 2 | 100 | 2 | 100 |  |
|  |  |  | >100% | 0 | 0 | 0 | 0 |  |
|  | 4 a 8 años | 3 | ≤100% | 3 | 100 | 3 | 100 |  |
|  |  |  | >100% | 0 | 0 | 0 | 0 |  |
|  | 9 a 13 años (varón) | 3 | ≤100% | 3 | 100 | 3 | 100 |  |
|  |  |  | >100% | 0 | 0 | 0 | 0 |  |
|  | 9 a 13 años (mujer) | 3 | ≤100% | 3 | 100 | 3 | 100 |  |
|  |  |  | >100% | 0 | 0 | 0 | 0 |  |
|  | 14 a 18 años (varón) | 3 | ≤100% | 3 | 100 | 3 | 100 |  |
|  |  |  | >100% | 0 | 0 | 0 | 0 |  |
|  | 14 a 18 años (mujer) | 3 | ≤100% | 3 | 100 | 3 | 100 |  |
|  |  |  | >100% | 0 | 0 | 0 | 0 |  |
| **Cobre** | 1 a 3 años | 2 | ≤100% | 1 | 50 | 2 | 100 |  |
|  |  |  | >100% | 1 | 50 | 0 | 0 |  |
|  | 4 a 8 años | 2 | ≤100% | 1 | 50 | 2 | 100 |  |
|  |  |  | >100% | 1 | 50 | 0 | 0 |  |
|  | 9 a 13 años | 2 | ≤100% | 1 | 50 | 2 | 100 |  |
|  |  |  | >100% | 1 | 50 | 0 | 0 |  |
|  | 14 a 18 años | 2 | ≤100% | 1 | 50 | 2 | 100 |  |
|  |  |  | >100% | 1 | 50 | 0 | 0 |  |
| **Flúor** | 1 a 3 años | 1 | ≤100% | 1 | 100 | 1 | 100 |  |
|  |  |  | >100% | 0 | 0 | 0 | 0 |  |
|  | 4 a 8 años | 1 | ≤100% | 1 | 100 | 1 | 100 |  |
|  |  |  | >100% | 0 | 0 | 0 | 0 |  |
|  | 9 a 13 años | 1 | ≤100% | 1 | 100 | 1 | 100 |  |
|  |  |  | >100% | 0 | 0 | 0 | 0 |  |
|  | 14 a 18 años | 1 | ≤100% | 1 | 100 | 1 | 100 |  |
|  |  |  | >100% | 0 | 0 | 0 | 0 |  |
| **Fósforo** | 1 a 3 años | 0 | ≤100% | - | - | - | - |  |
|  |  |  | >100% | - | - | - | - |  |
|  | 4 a 8 años | 1 | ≤100% | 1 | 100 | 1 | 100 |  |
|  |  |  | >100% | 0 | 0 | 0 | 0 |  |
|  | 9 a 13 años | 1 | ≤100% | 1 | 100 | 1 | 100 |  |
|  |  |  | >100% | 0 | 0 | 0 | 0 |  |
|  | 14 a 18 años | 1 | ≤100% | 1 | 100 | 1 | 100 |  |
|  |  |  | >100% | 0 | 0 | 0 | 0 |  |
| **Selenio** | 1 a 3 años | 1 | ≤100% | 1 | 100 | 1 | 100 |  |
|  |  |  | >100% | 0 | 0 | 0 | 0 |  |
|  | 4 a 8 años | 2 | ≤100% | 2 | 100 | 2 | 100 |  |
|  |  |  | >100% | 0 | 0 | 0 | 0 |  |
|  | 9 a 13 años | 2 | ≤100% | 2 | 100 | 2 | 100 |  |
|  |  |  | >100% | 0 | 0 | 0 | 0 |  |
|  | 14 a 18 años | 1 | ≤100% | 1 | 100 | 1 | 100 |  |
|  |  |  | ≤100% | 0 | 0 | 0 | 0 |  |
| Las celdas en gris representan los nutrientes para los cuales no existe UL reportado | | | | | | | |  |

**Figura suplementaria 1. Porcentaje de adecuación de UL de vitaminas según grupo de edad**


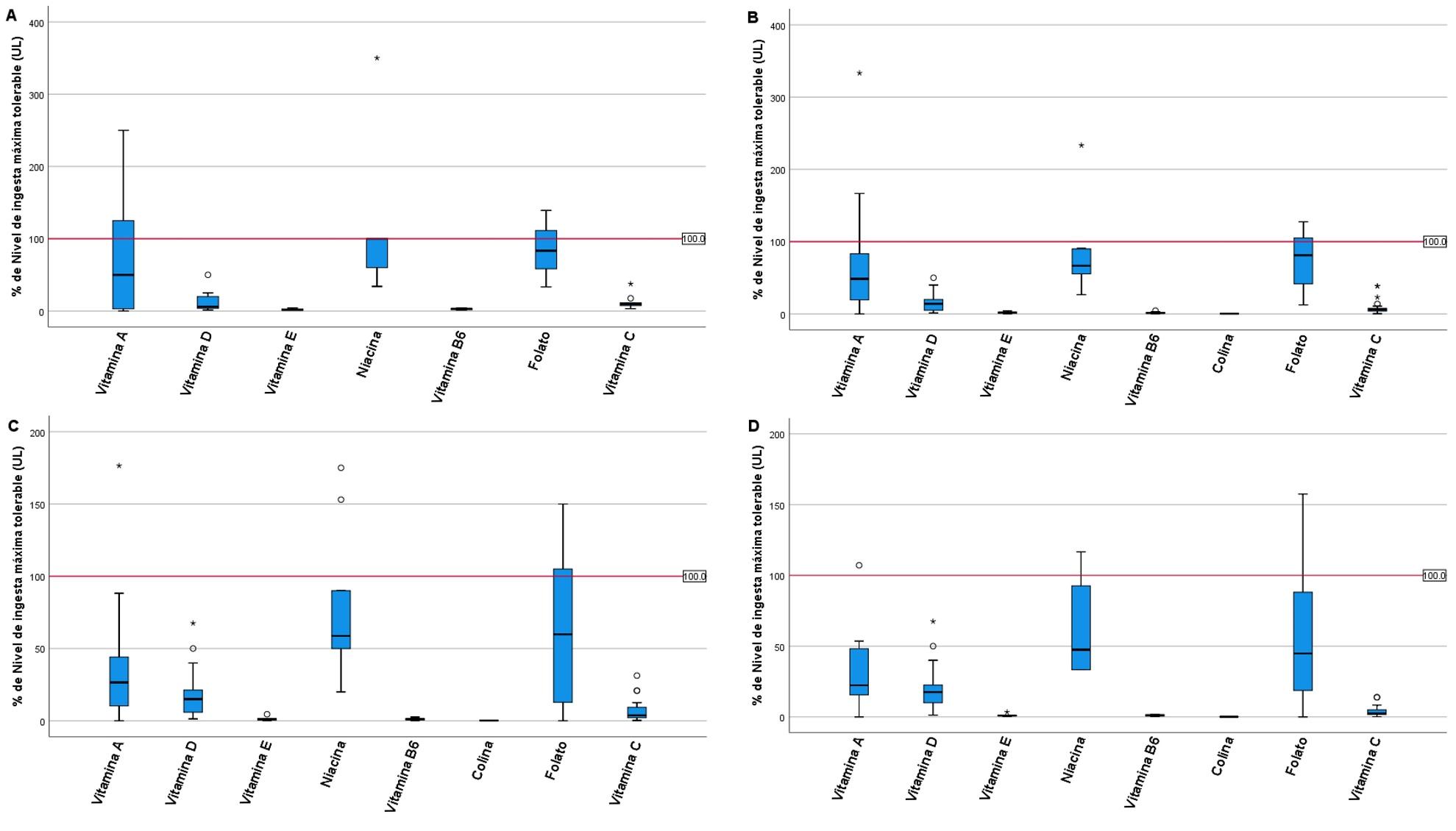


El número de suplementos evaluados para cada vitamina y grupo de edad varió según disponibilidad. La adecuación en porcentaje del UL de vitaminas se calculó por separado para niños de 1 a 3 años **(A)**, 4 a 8 años **(B)**, 9 a 13 años **(C)** y 14 a 18 años **(D)**. La línea roja horizontal representa el 100% del UL.

**Figura suplementaria 2. Porcentaje de adecuación de UL de minerales según grupo de edad**


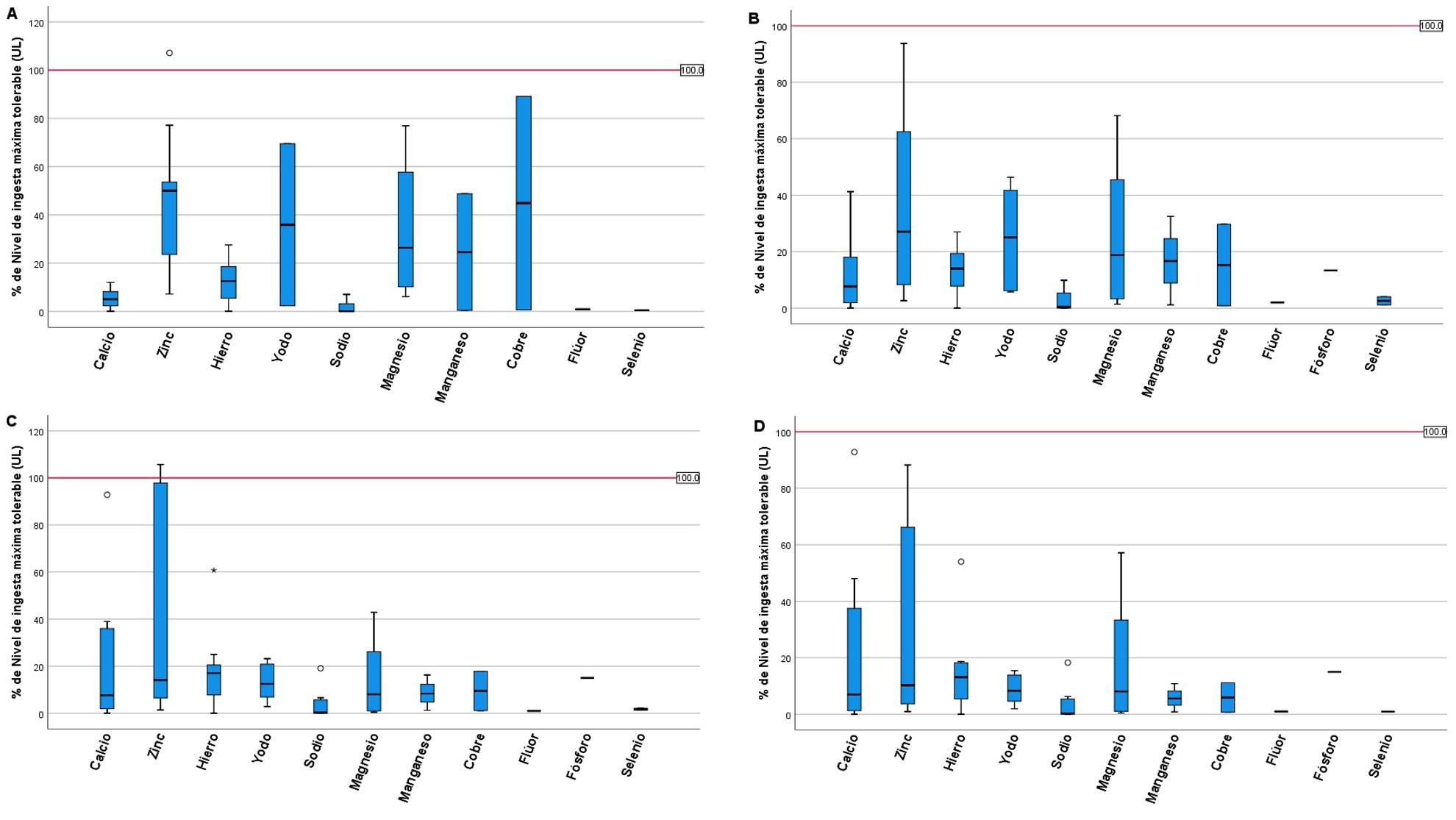


El número de suplementos evaluados para cada mineral y grupo de edad varió según disponibilidad. La adecuación en porcentaje del UL de minerales se calculó por separado para niños de 1 a 3 años **(A)**, 4 a 8 años **(B)**, 9 a 13 años **(C)** y 14 a 18 años **(D)**. La línea roja horizontal representa el 100% del UL.
