# Supplementary material for: Nutritional content of vitamin and mineral supplements aimed at children in the peruvian market: analysis of compliance with recommendations
Source: Rev Peru Med Exp Salud Publica. 2025 Mar 17;42(1):82–7. doi: 10.17843/rpmesp.2025.421.14256 (PMC12176023; doi:10.17843/rpmesp.2025.421.14256)
Supplement: Translation

# Translation: Contenido nutricional de suplementos de vitaminas y minerales dirigidos a niños en el mercado peruano: análisis de adecuación a las recomendaciones

**Authors:** Lorena Saavedra-Garcia, Anna Magdalena Gawlas, Antonella Quiroz-Macukachi, Hans Genmayel Donayre-Huamán, Raquel Idelsa Basurco-Olazabal, Jamee Guerra Valencia

**Affiliations:** 1 Grupo de Investigación en Nutrición Funcional, Carrera de Nutrición y Dietética, Facultad de Ciencias de la Salud, Universidad San Ignacio de Loyola, Lima, Perú.; 2 Carrera de Medicina, Facultad de Ciencias de la Salud, Universidad San Ignacio de Loyola. Lima, Perú.; 3 Facultad de Ciencias de la Salud, Universidad Privada del Norte, Lima, Perú.

**Keywords:** Suplementos Dietéticos, Niño, Vitaminas, Minerales, Ingesta Diaria Recomendada, América Latina

## Abstract

Se describió el contenido nutricional de suplementos dietéticos de vitaminas y minerales para niños en el mercado peruano y su adecuación respecto a las recomendaciones de ingesta dietética de referencia. Se realizó un estudio transversal que analizó la información declarada en el empaque de 34 productos expendidos en farmacias en 2022. Se calcularon las dosis diarias de cada micronutriente según las indicaciones del fabricante y se estimaron las adecuaciones respecto al aporte dietético recomendado (RDA) y nivel de ingesta máximo tolerable (UL) por grupo etario. Los micronutrientes más frecuentes fueron vitamina C (n=23), D (n=22), A (n=16), zinc (n=15) y B6 (n=14). En todos los grupos, la vitamina D, la tiamina, la riboflavina, el folato, la vitamina B12 y la vitamina C superaron las RDA. El zinc y cobre también excedieron las recomendaciones en algunos grupos. La vitamina A y el folato fueron los que más frecuentemente superaron el UL. Los hallazgos resaltan la necesidad de una mayor vigilancia sanitaria.

## INTRODUCCIÓN

A nivel mundial, la industria de los suplementos dietéticos alcanzó los USD 454 550 millones en 2021 con crecimiento en varias regiones ^1^. En Latinoamérica, el mercado de suplementos pasó del 3% en 1999 al 7% en 2017, siendo las vitaminas y minerales los más consumidos en Chile, Brasil y Perú ^2^. Este crecimiento también se observa en el segmento pediátrico, donde se estima que el mercado mundial crezca de 2 500 a 4 000 millones de USD en 2034 ^3^.

Las presentaciones atractivas y el marketing dirigido a los niños ^4^, junto con su disponibilidad sin prescripción médica, pueden generar la percepción de seguridad y fomentar su uso sin supervisión. Además, la falta de regulación específica sobre el contenido permite a los fabricantes usar libremente ingredientes y cantidades recomendadas, lo que conduce a la variabilidad en la composición ^5^^,^^6^, lo que, a su vez, puede llevar a dosis superiores a la ingesta máxima tolerable (UL, por sus siglas en inglés) ^6^^,^^7^.

Estudios previos han encontrado que los aportes de micronutrientes indicados en etiquetas de suplementos dietéticos suelen exceder las recomendaciones diarias (RDA por sus siglas en inglés) ^5^ e incluso las UL ^6^^,^^7^. Además, cuando se combinan el aporte dietético y suplementos, el exceso es más frecuente ^8^, sugiriendo un riesgo de sobreex.

En Estados Unidos hasta un 31,8% de hospitalizaciones asociadas a suplementos dietéticos se atribuye a efectos adversos y 10,2% a dosis excesivas ^9^ las cuales pueden generar múltiples eventos adversos como náusea, anorexia, hepatotoxicidad, segun el nutriente ^10^. Sin embargo, estos hallazgos provienen principalmente de países de altos ingresos, cuyos patrones alimentarios difieren de países de ingresos medios y bajos, donde la evidencia es limitada.

Investigar sobre el contenido de suplementos pediátricos en relación con las recomendaciones dietéticas es crucial, dado el creciente mercado y riesgos potenciales. El objetivo de este estudio es describir el contenido nutricional reportado en los suplementos dietéticos de vitaminas y minerales orientados a niños en el mercado peruano y comparar su aporte con las recomendaciones de ingesta diaria.

**MENSAJE CLAVE**

Motivación para realizar el estudio. La mayoría de los suplementos dietéticos de vitaminas y minerales se presentan en formatos atractivos para niños, como gomitas, y emplean técnicas de marketing dirigidas a ellos, lo que podría influir en el consumo y demanda de estos productos.

Principales hallazgos. Diversos suplementos excedieron las ingestas recomendadas, se destacó que aproximadamente la mitad de los productos que contenían vitaminas C, D, A y B6 excedieron los aportes dietéticos recomendados (RDA) / ingesta adecuada (AI) para el grupo de niños de 1 a 3 años.

Implicancias en salud pública. Aunque ninguna mediana de dosis de vitaminas y minerales superó el nivel máximo tolerable (UL), un cuarto de los productos excedió estos límites para vitamina A y folato en niños de 1 a 3 años, destacando la necesidad de una regulación más estricta.

## EL ESTUDIO

Se realizó un estudio observacional de corte transversal. Una de las autoras (LSG) seleccionó los productos entre el 15 y 22 de diciembre del 2022 en las páginas web de cinco farmacias con presencia nacional. Se accedió a la sección «Suplementos dietéticos para niños» y se identificaron 46 productos descritos como vitaminas y/o minerales (n=46). Se incluyeron aquellos con: (a) declaración de uso infantil o (b) presencia de estrategias de marketing orientadas a niños (lenguaje promocional, tipografía infantil, uso de personajes animados). Para productos con diferentes tamaños, se incluyó el de mayor contenido neto.

Se adquirieron 35 productos debido a la falta de disponibilidad de algunos al momento del envío. Tras verificar el nombre, la marca y la descripción, se excluyó uno descrito como suplemento probiótico.

Los productos fueron fotografiados para capturar cada lado del empaque, el contenido del inserto y cualquier instrumento de medición incluido. A partir de las fotos, se registraron en una hoja de Excel lo siguiente: presentación, país de origen, tipo (nutriente único o multimicronutriente), técnicas de marketing, lista de ingredientes, composición nutricional (contenido de vitaminas y/o minerales) y prescripciones declaradas (dosis, frecuencia y grupo etario).

Los valores nutricionales se estandarizaron según las unidades utilizadas por el Instituto de Medicina de los Estados Unidos (IOM, por sus siglas en inglés) ^11^. Se revisó el contenido de vitaminas: A, D, E, C, tiamina, riboflavina, niacina, ácido pantoténico, B6, biotina, folato, B12 y colina, y de minerales: calcio, zinc, hierro, yodo, sodio, potasio, magnesio, manganeso, cobre, flúor, fósforo y selenio. Para garantizar la calidad de los datos, la información fue ingresada dos veces por cuatro investigadoras (AMG, AQM, HGDH y RIBO). Se compararon los registros para resolver discrepancias mediante la revisión de fotos por un quinto investigador (JGV).

La variable de interés fue el contenido de nutrientes expresado como la proporción de la dosis diaria respecto a las ingestas dietéticas de referencia (DRI, por su sigla en inglés) de los grupos etarios: 1 a 3 años, 4 a 8 años, 9 a 13 años y 14 a 18 años ^11^. Se eligió esta forma de reporte debido a la falta de uniformidad en la declaración del contenido nutricional (por ejemplo: vitamina C 45 mg/2 gomitas; hierro 25mg/mL). Los grupos etarios se definieron segun el IOM, que establece recomendaciones diferenciadas por edad y, en algunos casos por sexo ^11^.

El IOM establece las DRI, que incluye la RDA o la ingesta adecuada (AI, por sus siglas en inglés) cuando no ha sido posible estimar la RDA, y el UL (Tabla suplementaria 1) ^11^. Se calculó el porcentaje del contenido de nutrientes en relación con la RDA o AI y con respecto al UL. Estos valores se obtuvieron dividiendo la dosis diaria del nutriente entre la DRI correspondiente. Cuando el denominador fue la RDA o AI, se calculó la adecuación respectiva; cuando fue el UL, se obtuvo el porcentaje del UL.

La dosis diaria se estimó según la prescripción del inserto o empaque, multiplicando el contenido nutricional por el número de dosis recomendadas al día para cada grupo etario. Para productos sin prescripción alguna (n=6), se utilizó el tamaño de porción declarada como la dosis diaria recomendada. En un producto con una única prescripción para todos los grupos etarios (n=1), se usó la misma indicación para todos los grupos. Estas decisiones se tomaron en base al principio de «consumidor razonable» propuesto por el Instituto Nacional de Defensa de la Competencia y de la Protección de la Propiedad Intelectual ^12^.

En productos con prescripciones expresadas en rangos, (ej. «consumir de 1 a 3 tabletas por día»), se utilizó el valor más alto para estimar el mayor consumo posible. Finalmente, la adecuación de RDA o AI y el porcentaje del UL se calculó para cada nutriente, grupo etario y producto según las fórmulas:



Dado que el IOM establece recomendaciones diferenciadas por sexo para algunos micronutrientes en los grupos etarios de 9 a 13 y 14 a 18 años, los cálculos en estos escenarios también se realizaron por separado según el sexo.

No se realizó estos cálculos para el cobalto (n=1) por la falta de DRI.

Se empleó STATA versión 17.0 para análisis descriptivos, con frecuencias absolutas y porcentajes para variables categóricas. Las adecuaciones de RDA o AI y los porcentajes de UL se reportaron mediante mediana, percentiles 25 y 75 y los valores mínimos y máximos, debido a la alta dispersión en los datos ^5^, evitando así los sesgos por valores atípicos. Con SPSS versión 27.0 se construyeron diagramas de cajas y bigotes para visualizar las adecuaciones de RDA o AI y UL por grupo etario, fijando en el eje Y el 100% como línea de contraste.

El estudio se basó en la información declarada en el empaque de los suplementos, por lo que no representó un riesgo para los seres humanos y no requirió aprobación de un comité de ética institucional.

## HALLAZGOS

Se analizaron 34 productos. Los nutrientes más frecuentes fueron vitamina C (n=23), vitamina D (n=22), vitamina A (n=16), zinc (n=15) y vitamina B6 (n=14), mientras que los menos comunes fueron vitamina K, colina, cobre, cobalto, flúor, fósforo, selenio y molibdeno (Tabla suplementaria 2). Aproximadamente la mitad de suplementos fueron de manufactura nacional y el 76,5% era multimicronutriente (≥2). La presentación más frecuente fue en gomitas; y 82,4% no requerían reconstitución. Se destaca que un 58,8% no presentó lista de ingredientes y un 70,6% usó técnicas de marketing dirigidas a niños. Además, el 17,7% no declaró prescripción y el 29,4% no especificó dosis según grupo etario (Tabla 1).

**Tabla 1:** Características de los suplementos dietéticos analizados (n=34).

Características		n	%
Origen del producto			
	Nacional	16	47,1
	Internacional	18	52,9
Presentación			
	Gomitas	13	38,3
	Jarabe	8	23,5
	Tabletas efervescentes	3	8,8
	Polvo	3	8,8
	Otro ^a^	7	20,6
Presencia de octógono			
	Sí	5	14,7
	No	29	85,3
Presencia de registro sanitario			
	Sí	33	97,1
	No	1	2,9
Requiere reconstitución			
	Sí	6	17,7
	No	28	82,3
Declara lista de ingredientes			
	Sí	14	41,2
	No	20	58,8
Cantidad de nutrientes del producto			
	Solo uno	8	23,5
	Dos o más	26	76,5
Presenta técnicas marketing para niños			
	Sí	24	70,6
	No	10	29,4
Declara al menos una prescripción			
	Sí	28	82,3
	No	6	17,7
Declara grupo etario para prescripción			
	Sí	24	70,6
	No	10	29,4

a Incluye solución oral en gotas, tabletas recubiertas y tabletas masticables.

El porcentaje de adecuación de la RDA o AI reveló que, en niños de 1 a 3 años la mediana de la dosis de tiamina, riboflavina, vitamina B6, folato, B12 y vitamina C superó las recomendaciones. En el grupo de 4 a 8 años se observó lo mismo para vitamina D, tiamina, riboflavina, niacina, ácido pantoténico, vitamina B6, folato, vitamina B12 y vitamina C. En los grupos de 9 a 13 y 14 a 18 años las medianas de vitamina D, riboflavina y vitamina B12 superaron las recomendaciones (Figura 1).

**Figura 1:** Porcentaje de adecuación de aportes dietéticos recomendados (RDA) o ingesta adecuada (AI) de vitaminas según grupo de edad.

El número de suplementos evaluados para cada vitamina y grupo de edad varió según disponibilidad. La adecuación en porcentaje del RDA o AI de vitaminas se calculó por separado para niños de 1 a 3 años (A), 4 a 8 años (B), 9 a 13 años (C) y 14 a 18 años (D). La línea roja horizontal representa el 100% de la adecuación para RDA o AI.

Respecto a los minerales, en el grupo de 1 a 3 años las medianas de dosis de zinc y cobre superaron las recomendaciones, mientras que para el grupo de 4 a 8 años solo fue el cobre . En los grupos de 9 a 13 y 14 a 18 años, ninguna mediana de dosis de minerales superó las RDA o AI (Figura 2 y Tablas Suplementarias 3, 4 y 5).

Aunque ninguna mediana de dosis de vitaminas ni minerales superó el UL, el 25% de las dosis excedió el UL para vitamina A y folato en 1 a 3 años y para folato en 4 a 13 años (Figuras suplementarias 1 y 2).

**Figura 2:** Porcentaje de adecuación de aportes dietéticos recomendados (RDA) o ingesta adecuada (AI) de minerales según grupo de edad

El número de suplementos evaluados para cada mineral y grupo de edad varió según disponibilidad. La adecuación en porcentaje del RDA o AI de vitaminas se calculó por separado para niños de 1 a 3 años (A), 4 a 8 años (B), 9 a 13 años (C) y 14 a 18 años (D). La línea roja horizontal representa el 100% de la adecuación para RDA o AI.

## DISCUSIÓN

Los resultados revelan aspectos importantes sobre el contenido de vitaminas y minerales en suplementos dietéticos para niños en el mercado peruano. Se observó predominancia de suplementos multimicronutrientes y presentación en gomitas, un formato atractivo y fácil de consumir para los niños. Entre los nutrientes analizados, las vitaminas hidrosolubles como el complejo B y la vitamina C, así como las liposolubles A y D, presentaron dosis que frecuentemente superaron las RDA o AI. Entre los minerales, el zinc destacó por superar las recomendaciones en niños de 1 a 3 años. Aunque las medianas de dosis de los nutrientes no superaron el UL, ciertos productos excedieron estos límites, especialmente para la vitamina A y el folato en los grupos etario más jóvenes.

Estos hallazgos coinciden con estudios realizados en otros países. En Canadá, se reportó que la mayoría de los suplementos para niños contenía dosis de nutrientes superiores a las recomendaciones diarias ^5^. En Estados Unidos, casi 70% de los suplementos dietéticos presentaba cantidades de nutrientes iguales o superiores a las RDA ^13^. Esta situación puede estar impulsada por estrategias como el marketing. En este estudio, la mayoría de los productos utilizaban marketing dirigido a niños, lo que podría influir en la demanda y el consumo de suplementos, como se ha documentado en estudios previos ^14^. Esta promoción, sumada a las presentaciones de fácil consumo como gomitas, y al alto contenido de algunos nutrientes hallado en ciertos productos, puede conducir a que los consumidores excedan las recomendaciones de nutrientes. Esto es particularmente preocupante, considerando que instituciones como la Academia de Nutrición y Dietética de Estados Unidos desaconsejan el uso de suplementos en niños con dietas equilibradas que cubren sus requerimientos ^15^.

Por otro lado, el uso de suplementos multimicronutrientes, aunque puede facilitar la ingesta de múltiples nutrientes simultáneamente, plantea interrogantes sobre posibles interacciones que afecten la absorción y la bioactividad de los nutrientes. Por ejemplo, el calcio y el hierro pueden interferir con la absorción de zinc y magnesio cuando se incluyen en la misma formulación ^16^. Esto resalta la importancia de evaluar la composición y la combinación de nutrientes al desarrollar y recomendar suplementos.

Otro hallazgo relevante es la presencia de suplementos con dosis que superan los UL, como en el caso de la vitamina A. La toxicidad por vitamina A puede provocar efectos adversos graves como hepatotoxicidad y alteraciones visuales ^10^. El folato también se encontró en dosis superiores al UL. Aunque estudios han señalado que, en niños la ingesta puede superar este límite sin efectos adversos identificados, dosis elevadas pueden enmascarar deficiencias de vitamina B12 y agravar neuropatías ^17^. En el caso del zinc, su exceso puede causar náuseas, vómitos, deficiencias de cobre y problemas de crecimiento ^18^, además de un mayor riesgo de anemia ^19^.

Este estudio presenta limitaciones. La revisión del contenido de nutrientes se basó en la información declarada en los empaques, sin verificación mediante análisis de laboratorio, lo que podría introducir sesgos si la información declarada no es precisa. Además, los productos se seleccionaron en un período específico del año 2022 y en las secciones «Suplementos dietéticos para niños» de farmacias en línea, lo que pudo excluir productos indicados tanto para adultos como para niños disponibles en otras secciones y, posiblemente no representar, la totalidad del mercado. Finalmente, no se evaluó el consumo de suplementos, por lo que no es posible confirmar que las dosis reflejen el consumo real en la población.

Es relevante desarrollar nuevas investigaciones que analicen el impacto del consumo de suplementos en la ingesta total de nutrientes en niños peruanos, así como estudiar las interacciones entre nutrientes en suplementos multimicronutriente. En paralelo, se requiere educar a los padres sobre su consumo para garantizar seguridad y eficacia. En tal sentido, implementar un etiquetado riguroso, como el establecido por la FDA ^20^, y contar con pronunciamientos de autoridades regulatorias y de salud como lo establece la Academia de Nutrición y Dietética de Estados Unidos ^15^^)^ es fundamental.

En conclusión, el análisis de suplementos de vitaminas y minerales para niños en el mercado peruano muestra variabilidad en la adecuación de sus dosis. Varios productos superan las ingestas recomendadas y, en algunos casos, los niveles máximos tolerables de vitamina A y folato. Estos hallazgos destacan la necesidad de vigilar estos productos y garantizar un etiquetado claro.
